# Sensitive listening of women-mothers caring for children with cancer about self-care

**DOI:** 10.1590/0034-7167-2025-0027

**Published:** 2026-01-09

**Authors:** Dociana Erica Cabral Formigosa, George Pinheiro Carvalho, Lucrecia Aline Cabral Formigosa, Hosana de Nazaré Miranda de Carvalho, Joana Dulce Cabral Formigosa, Renato da Costa Teixeira, Marcia Helena Machado Nascimento, Maria Goreth Silva Ferreira

**Affiliations:** I Universidade do Estado do Pará. Belém, Pará, Brazil.; II Universidade do Estado do Pará. Santarém, Pará, Brazil.

**Keywords:** Maternity, Child, Self-Care, Neoplasms, Nursing Care, Maternidades, Niño, Autocuidado, Neoplasias, Atención de Enfermería

## Abstract

**Objectives:**

to understand the experiences of mothers who care for children with cancer regarding self-care.

**Methods:**

descriptive, qualitative research using the Creative Sensitive Method. Data collection took place at the outpatient clinic of a children’s cancer hospital in Belém, Pará, with 16 participants. These sessions were conducted through creativity and sensitivity dynamics in two sessions. These sessions were recorded, transcribed, and analyzed using French discourse analysis, based on Paulo Freire’s theoretical framework.

**Results:**

three main topics emerged: 1) “Impacts on caregiving mothers’ health”, with emotional, psychological, and physical impacts subtopics; 2) “Dedication to child care and its impact on family dynamics”, including the family relationships subtopic; 3) “Institutional support and public policies”.

**Final Considerations:**

caregiving mothers face significant impacts on their physical and emotional health, and family dynamics, requiring the implementation of care strategies that consider their experiences and improve their quality of life.

## RESUMEN

**Objetivos:**

comprender las experiencias de autocuidado de madres que cuidan a niños con cáncer.

**Métodos:**

investigación cualitativa descriptiva mediante el Método Creativo Sensible. La recolección de datos se realizó en la consulta externa de un hospital oncológico infantil en Belém, Pará, con 16 participantes. Estas sesiones se realizaron mediante dinámicas de creatividad y sensibilidad en dos sesiones. Estas sesiones se grabaron, transcribieron y analizaron mediante análisis del discurso francés, con base en el marco teórico de Paulo Freire.

**Resultados:**

se desarrollaron tres temas principales: 1) “Impactos en la salud de la madre cuidadora”, con subtemas sobre impactos emocionales, psicológicos y físicos; 2) “Dedicación al cuidado infantil y su impacto en la dinámica familiar”, incluyendo el subtema sobre relaciones familiares; 3) “Apoyo institucional y políticas públicas”.

**Consideraciones Finales:**

las madres cuidadoras enfrentan impactos significativos en su salud física y emocional, así como en la dinámica familiar, lo que requiere la implementación de estrategias de cuidado que consideren sus experiencias y mejoren su calidad de vida.

## INTRODUCTION

Cancer is a disease as old as humanity, recognized as one of the main global health challenges, raising significant concerns regarding prevention, early diagnosis, treatment, and rehabilitation. Recent data indicate that in countries with a low Human Development Index (HDI), a 112% increase in cancer cases is projected, while countries with a medium HDI may experience an 86% increase between 2012 and 2035^(1)^.

In Brazil, approximately 704,000 new cases of the disease are expected to be recorded in the 2023-2025 triennium, excluding non-melanoma skin cancer. Of these cases, 7,930 will occur in children and adolescents aged 0 to 19, with 4,230 males and 3,700 females. In this context, the highest cancer incidence rates stand out, with 3,310 new cases in the Southeast and 2,130 in the Northeast, while the South, Central-West, and North will have significantly lower estimates, with 1,180, 660, and 650 cases, respectively^(2)^.

When hospitalizing pediatric patients, the presence of a companion is necessary, and health facilities must adapt to the presence of parents or guardians during the hospitalization period, guaranteeing them equal access^(3)^. In general, it is the mother who most closely accompanies her child during the process of illness and hospitalization, which is an emotionally and physically exhausting experience for women-mothers who care for them, and can lead to numerous health problems if there is no adequate support^(4)^.

In this study, the term “caregiving women-mothers” was used to reflect the complex role of a woman who, in addition to caring for their children in traditional ways, also take significant and often challenging responsibilities related to serious health conditions such as cancer. This multifaceted role requires comprehensive support and a sensitive approach to effectively meet their needs^(5)^.

The literature indicates that the impact of childhood cancer diagnosis and treatment extends beyond children, significantly affecting caregivers due to the physical, psychological, emotional, and social burden. It is known that uncertainty regarding children’s treatment and prognosis, coupled with feelings of helplessness, profound sadness, and physical and emotional exhaustion, can lead to the development of depression, directly impacting various aspects of caregivers’ health, such as sleep disorders, changes in eating patterns, intense fatigue, and immunocompromised health, in addition to negatively impacting their social, emotional, and relationship lives^(6)^.

However, gaps persist in the comprehensive approach to these mothers in the context of childhood cancer treatment, justifying the need for this study, which focuses on understanding the repercussions of this process on these women’s lives, with an emphasis on how they perceive and experience self-care. In this regard, it is essential to consider that caregiving women-mothers’ health is a central element in the quality of care provided to children undergoing cancer treatment. Therefore, it is essential that they also receive attention and support throughout their children’s treatment journey. This understanding seeks to inform more sensitive multidisciplinary care and support practices, in addition to contributing to the formulation of interventions that consider the needs and specificities of these mothers, aiming to improve their quality of life and, consequently, that of the child undergoing treatment^(7,8)^.

## OBJECTIVES

To understand the experiences of women-mothers who care for children with cancer regarding self-care.

## METHODS

### Ethical aspects

The research complied with the ethical precepts of Resolution 466/2012 of the Brazilian National Health Council, which regulates research involving human subjects, and was approved by the Research Ethics Committee of the *Escola de Enfermagem “Magalhães Barata”*, *Universidade do Estado do Pará*. Participants’ ethical consent was obtained by signing the Informed Consent Form. To maintain anonymity, participants were identified throughout the text by alphanumeric codes (M1...M16).

#### Theoretical framework

The investigation of caregiving women-mothers’ experiences was based on Paulo Freire’s critical-reflective theoretical framework, operationalized by the Creative Sensitive Method (CSM), since this favors sensitive listening, dialogicity and collective construction of knowledge, allowing a deep understanding of meanings and senses attributed by participants to their experiences, considering the biopsychosocial aspects^(9-11)^.

The theoretical framework used was Paulo Freire’s critical and reflective approach, which establishes educational practice as an act of liberation from the oppressive constraints of society, enabling them to become active agents in the construction of knowledge and the change of a given reality. The uniqueness of Paulo Freire’s method emerges in issues related to individuals’ consciousness, in which a new perspective on reality is generated by critical thinking, as well as community consciousness to deal with common extreme situations^(9,10)^.

Paulo Freire’s theory emphasizes that dialogue plays a prominent role in the educational process, which, to be authentic, must be based on equality and mutual respect, with active and reflective participation from both students and educators, in the pursuit of action and emancipation. Thus, dialogicity, intertwined with lovingness, must be present at all moments of this process, from the choice of content to generating topics and method, which makes it possible to build an ethical, social and critical pedagogy capable of bringing liberating awareness of the oppression still in force in society^(9)^.

#### Study design

This is descriptive research, with a qualitative approach, based on CSM, which is based on the triad of group discussion, participant observation and creativity-sensitivity dynamics^(11)^. In this regard, the method is suitable for the object of study because it is based on the principle of dialogicity, which emphasizes the inseparability of human beings from their history and recognizes language as an essential instrument of social interaction^(12)^. Furthermore, Paulo Freire’s critical-reflective framework allows participants to express their ideas and opinions freely, promoting a de-oppression practice mediated by freedom of thought, encouraging creativity and multiple forms of expression, especially the body’s senses and sensitivity through interactive dynamics^(9,13)^.

#### Methodological procedures

##### Study setting

The study was conducted in the outpatient clinic of a children’s cancer hospital in the municipality of Belém, Pará state. The setting was chosen because it is a High Complexity Oncology Unit and the only public hospital in Pará offering specialized cancer treatment for children and adolescents aged 0 to 19 years^(14)^.

##### Participants

Sixteen women-mothers, caregivers of children diagnosed with and undergoing cancer treatment at the hospital’s outpatient clinic, participated in the study. Inclusion criteria comprised mothers of children diagnosed with cancer aged 0 to 11 years, 11 months, and 29 days^(15)^ and undergoing treatment for at least six months at the study hospital. Mothers under 18 years of age, those who were illiterate, or those with cognitive impairment and/or comorbidities that prevented data collection were excluded.

##### Data production

Data collection took place from January to February 2024, through creativity-sensitivity dynamics, which are the central pillars of CSM. Participants were randomly divided into two subgroups of eight caregivers, each with the tree of knowledge and body-knowledge dynamics selected for data collection. The meetings were recorded in video and audio format on smartphones and later transcribed.

The dynamics operationalization involved five stages: 1) organization of the environment and materials to be used in the artistic productions; 2) presentation of the research objective and reading of the Informed Consent Form and the Image and Sound Usage Agreement, in addition to completing the sociodemographic profile form; 3) explanation of the dynamics and its objectives, as well as the distribution of materials for artistic construction and presentation of the question generating the debate; 4) presentation of individual artistic production, followed by the collective analysis of individual experiences; 5) collective analysis, synthesis of experiences, and data validation. It is worth noting that the activities were carried out by a principal researcher, two assistant researchers, and a psychologist, aiming to provide psychological support to those who needed it.

The tree of knowledge dynamic is based on the collective artistic production of a tree, in which participants establish analogies between the parts that compose it and the research object proposed by the subject-researcher, also considering the question generating the debate^(16)^. This approach fostered dialogue among participants, revealing the meanings of the self-care process, linked to the experiences they had during their children’s diagnosis and treatment. The question prompting the discussion was formulated as follows: just as a tree needs natural elements to grow strong and healthy, producing flowers and fruit, what do you consider important for the self-care process during your child’s treatment?

The body-knowledge dynamic is developed with artistic production by participants, using certain resources, such as posters, colored pencils and pens, being mediated by the drawing of a human body to relate to healthcare, whether for oneself or for others^(17)^. The activity was developed based on the following debate-generating question: how does a child’s illness and hospitalization process reflect on their body and health?

##### Data analysis

The analysis of the transcribed material was conducted using French discourse analysis, using texts generated through creativity and sensitivity dynamics. This methodology is supported by linguistic materiality and explores meaning production during reading. This allows us to capture meanings that go beyond words, considering pauses and interruptions that affect discourse and are interconnected with the emotional and cognitive tensions present in the narratives^(18)^. Furthermore, this analysis is divided into three stages: pre-discursive analysis; discursive analysis; and post-discursive analysis^(19)^.

In pre-discursive analysis, the text *corpus* was defined according to caregiving women-mothers’ discourses. Discursive analysis employed four phases, expressed by data transcription with a skim reading and identification of topics, discursive object analysis, in which the originating meanings are understood, discursive process analysis, with the attribution of meanings based on the relationship between verbal and non-verbal discursive activities, and development of discursive typologies. Finally, post-analysis was based on analysis contextualization, interpretation, and presentation based on its relationship with Paulo Freire’s theoretical framework^(19,20)^.

### RESULTS

#### Participant characterization

Sixteen caregivers participated in the study, five of whom lived in the Belém metropolitan area and 11 lived in other municipalities, placing them a significant distance from the location where their children receive treatment. Ages ranged from 22 to 47. Eight women had completed high school, followed by six with elementary school, and only two had higher education. The predominant marital status was married, with eight participants in this status, two in a common-law union, while three identified themselves as divorced and three as single. The majority of participants, totaling 11, were unemployed, dedicating themselves to domestic activities, and ten of them had a family income of only one minimum wage. Regarding children, most women had two children, with a mean age of 7, and the mean time since diagnosis was three years.

The data are initially presented through caregiving women-mothers’ artistic productions, who express their subjective experiences in the context of their children’s cancer care. The impacts of this condition on multiple spheres of these women’s lives, including emotional, psychological, physical, social, and family aspects, are then discussed, providing an integrated and in-depth understanding of their experiences.

#### Tree of knowledge dynamics

**Figure 1:**
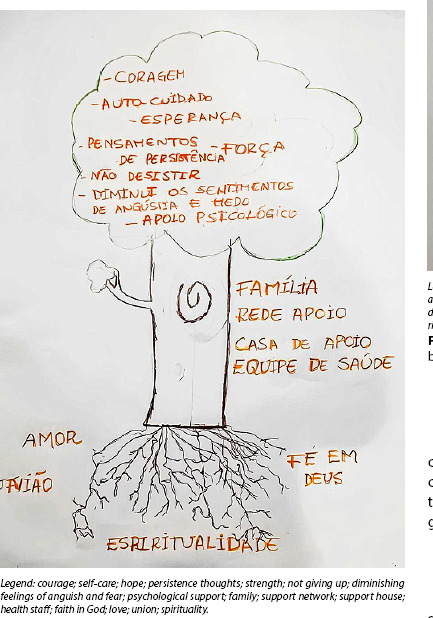
Artistic production by women-mothers during the tree of knowledge dynamics, Belém, Pará, Brazil, 2024

During a meeting, participants developed an artistic production and shared their experiences with diagnosis and treatment of their child with cancer in a group setting. Following the discussion question, they described the elements they considered important for self-care during their children’s treatment, as shown in Figure 1.

#### Body-knowledge dynamic

**Figure 2:**
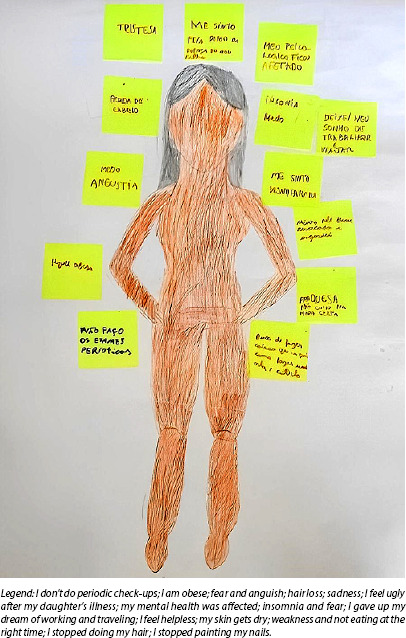
Artistic production by caregiving women-mothers during the body-knowledge dynamic, Belém, Pará, Brazil, 2024

From this dynamic, an artistic production was created, and a group discussion arose about the health impacts of women-mothers caring for children with cancer and how the self-care process unfolds. Based on the discussion question, participants shared their experiences regarding the health-disease process, including signs of illness and treatment, as illustrated in Figure 2.

#### Impacts on caregiving women-mothers’ health

The experiences of mothers caring for their children reveal a complex scenario, where the demands of caring for a child with cancer profoundly impact the dynamics of their lives. Furthermore, this impact is multifaceted, affecting various spheres, including caregiving women-mothers’ personal, professional, and emotional lives.

#### Emotional and psychological impacts

Caring for a child with cancer is arduous, intense, and demands specific attention due to the specificities of treatment and its consequences. Mothers constantly deal with an overload of tasks, especially when it comes to providing comprehensive care for their child, which ultimately impacts their caregiving process. Analysis of participants’ statements reveals common patterns of neglected self-care, as their reports point to prioritizing childcare over their own priorities and health, highlighting the relationship with fatigue and exhaustion.

*After going through everything* [illness], *I think I became more tired, more carefree about myself, you know?* [...]. (M1)

*But my biggest concern is with her and I end up leaving myself aside* [...]. (M2)

[...] *I think that since I focused so much time on taking care of her* [daughter undergoing treatment]*, we end up neglecting ourselves more* [...]. (M5)

*In these four years, the only doctor I go to is the gynecologist, and that’s when I feel any discomfort* [...]. (M11)

[...] *like taking care, not taking care. The only care I have, like, is just asking God to protect me* [...]. (M16)

The constant prioritization of their children’s needs reverberates in the depletion and exhaustion not only physical, but also psychological and emotional, of caregiving women-mothers, resulting in a state of chronic fatigue, which can lead to the development of psychiatric disorders. Participants’ statements reflect a discursive practice strongly marked by distress, anxiety, and depression, which are effects of the experience of caring for a child with cancer. Thus, the mothers construct their identities in their discourse as exhausted caregivers, overwhelmed by the responsibility of maintaining their children’s lives.

*I also got like this; my husband pulled me out of a rope after my daughter’s diagnosis* [...]. (M5)

[...] *I tried to kill myself after my daughter’s diagnosis*. (M9)

[...] *we feel a tremendous amount of anxiety. You’re constantly there, as if your child’s lifeline could snap at any moment. So, there are a lot of ups and downs, and then you end up really taking it out on food. Some end up going that way, others become really depressed. I went back and forth from depression so quickly, because at the same time I was at rock bottom, but I knew he needed me. And then, through faith, we can do this* [...]. (M12)

*I have anxiety* [...]. (M4)

[...] *I have that too* [anxiety]. (M7)

Furthermore, the graphic production in the tree of knowledge dynamic confirmed the importance attributed to spirituality and faith, highlighted in the statements as essential resources during their children’s illness. Participants cited expressions such as “faith in God” and “spirituality”, revealing that these elements act as emotional support and strengthen daily coping. On the other hand, participants highlighted the influence of the support network, including the “Support House”, family members, and health staff as elements that help minimize the impacts of intensive care for children undergoing cancer treatment.

#### Physical impacts

A child’s diagnosis causes significant physical strain for caregiving women-mothers, who take primary responsibility for caring for their sick child. Participants’ statements reveal how treatment impacts their physical needs, often neglected for the sake of their children’s well-being. These expressions are embedded in a context of health and illness, reflecting ideologies related to the body and femininity. Furthermore, mothers’ subjectivity is shaped by the experience of illness, revealing a fragile self that faces the bodily and emotional changes brought about by their children’s treatment.

[...] *I have a lot of headache, migraine*. (M6)

[...] *I gained weight. When she started her treatment, I weighed 48* [kg]*. Today I weigh 60* [kg]. (M12)

[...] *when I get stressed, anxious about something, purple spots start to appear*. (M15)

[...] *my hair was really long, it fell out a lot, I cut it, but I couldn’t get it back, because it seems like when she does chemo especially, it seems like when I get home and put my hand in, wow, a lot of hair comes out*. (M16)

[...] *unfortunately, there’s a lot of stuff to do at home and we don’t have time for anything. When we finish, it’s already ten, 11 o’clock at night, we have to sleep, because I come to the hospital three, four times a week with her, so I don’t have much time for myself*. (M16)

#### Dedication to child care and its impact on family and social dynamics

The debate, mediated by artistic production, paints a picture of how women face significant challenges due to the demands of daily childcare, encountering factors related to migration, family and professional experiences, cultural aspects, linguistics, religious beliefs, and financial issues. Each of these dimensions is reflected in social structures and processes that shape maternal experiences during children’s care process. Understanding the impacts of this reality and its influences on their health and well-being is essential, as well as understanding the potential strategies adopted to cope with these experiences.

#### Family relationships

Based on the discourses, it was found that caring for sick children affects family dynamics, including relationships with spouses, other children, and close family members, in addition to the financial impact. The discursive practices of these caregiving mothers reveal how a child’s hospital treatment interferes with their daily lives and family relationships. This discursive formation is permeated by ideological notions of motherhood, sacrifice, and guilt, as well as the overload of activities they perform, reinforcing the social perspective that values mothers’ selflessness.

[...] *my other son* [the brother of the child undergoing treatment] *complains a lot about this. He says, “Mom, you don’t pay attention to me”*. (M16)

*My other son also complains a lot. He says, “Mom, you give her* [the child undergoing treatment] *a lot more attention. It’s just her, the hospital and her, it’s home and her”. He only goes to school, like, he doesn’t go out because I don’t have much time with him*. (M2)

In addition to influencing social relationships, the therapeutic process of a child with cancer also impacts the family’s livelihood due to expenses with transportation, food, and accommodation for mothers in the treatment city, in the case of those who come from other regions, among other factors, which end up overloading the family budget and influencing the life dynamics of those involved.

[...] *but I literally abandoned everything and we sold everything we had. We sold our house, our car, everything we could so that I wouldn’t have to go back to work during this period so I could continue my treatment, which, despite being through the SUS, our expenses are high* [...]. (M5)

[...] *and there was a problem, due to financial issues, We, in fact, kept what was left and it was all for him* [...]. (M9)

#### Institutional support and public policies

Mothers’ statements reflect their distress and vulnerability, often expressing a request for support, which can be interpreted as a demand for institutional and public health policies that recognize the emotional impact of their children’s illness on mothers’ health. This is essential for promoting their well-being, which, in turn, improves their quality of life during their children’s treatment. It is important to emphasize that mothers’ mental health affects not only their own living conditions but also the quality of care they provide to their children.

[...] *sometimes, even here at the hospital, we’re labeled as stressed, but we’re not. We’ve already been through so many situations. It seems like people like that have no empathy, they don’t put themselves in someone else’s shoes*. (M3)

[...] *I think there should be psychological support for mothers, you know? I think it would help a lot*. (M1)

## DISCUSSION

The polyphony of the discourse of these caregiving mothers reveals that, when their child becomes ill, they dedicate themselves entirely to caring for the child, putting their own needs second. In this journey of extreme dedication to their children, they neglect their own health, reflecting a process of self-forgetfulness, in which mothers’ well-being is subjugated to child demands. Thus, by taking over as a caregiving mother, these women endure the pain and constantly dedicate themselves to the child, which prevents them from recognizing that they also need care^(21)^.

Self-neglect, present in the discourses, indicates changes in identity and self-perception during the process of caring for others, impacting women’s self-image and self-esteem, as well as their lifestyle and vulnerability to health problems^(22)^. Immersion in the dynamics of caring for a hospitalized child is marked by responsibilities and uncertainties that generate a feeling of constant risk, in line with Freire’s thinking, as mothers do not find space to distance themselves from the situation in which they are inserted and are unable to distance themselves to critically reflect on their condition and, consequently, there is no paradigm shift^(23)^.

The daily lives of these mothers are marked by continuous stress, constant worry and lack of rest, which negatively impacts their physical condition and quality of life^(24)^. Participants report symptoms such as headaches, migraines, and bruising, illustrating the association between stress and the body’s reactions, reflecting the mental and emotional overload they face^(25)^. Additionally, mothers mention negative physical changes, such as weight gain and hair loss related to chemotherapy medications, which affect their self-esteem and self-image, influenced by social standards of beauty and femininity^(26)^.

The discursive formations denote suicide attempts resulting from extreme distress and exhaustion, reflecting the critical level of pain and despair experienced upon their children’s diagnosis. The language used by women reflects the rupture in their subjectivity, as cancer carries the stigma of being a death sentence, signifying a tragic outcome for the child, leading to distress and severe psychological and emotional crises^(27)^.

Symptoms of psychiatric disorders reflect the significant impact on caregivers’ physical and mental health, quality of life, and immune system, exacerbating the possibility of developing comorbidities, whether acute or chronic. Furthermore, caregiver illness implies a deficit in the quality of care provided as well as in the continuity of activities carried out in the personal, family, and/or professional spheres^(28)^.

The findings indicate that at certain times, participants use religiosity and spirituality as a strategy to alleviate distress, control emotions, and find strength during the treatment of a child with cancer. Religiosity helps relieve stress, promotes psychological balance, and serves as a coping mechanism and emotional foundation. Therefore, standardizing care guidelines and practices is essential, as well as implementing strategies to address psychiatric and psychosomatic disorders, aiming to empower caregivers and reduce their burden, resulting in a reduction in the negative impact on their health and well-being^(29)^.

Based on participants’ statements, dialogue is identified as a way to foster care, serving as a tool for awareness and social transformation that proposes education as a reflective and liberating practice, based on empathy and mutual recognition of experiences, known as liberatory education. This approach encourages the search for support, community organization, and the strengthening of bonds to combat emotional and social oppression. Furthermore, dialogue with mothers allowed them to express their emotions, fears, and insecurities related to children’s treatment, fostering recognition of their own needs and the search for the best way to care for themselves while caring for their children^(9)^.

In this context, nurses play a key role in overcoming oppression, promoting care strategies that foster the humanization of care and the building of therapeutic bonds of trust based on Freirean precepts. These strategies lead caregiving women-mothers to recognize their own condition and act to transform it. In other words, sensitive listening and dialogue emerge as awareness-raising strategies that can lead them to rethink the balance between caring for others and caring for themselves and recognize the importance of their own well-being, allowing them to be fully valued^(30)^.

Family dynamics are completely affected as a result of children’s cancer treatment, since mothers dedicate themselves to activities linked to the sick child, such as hospitalization and hospitalization, medical appointments, medication administration, among others, which can lead to withdrawal from other family, social and marital demands^(31)^. The speeches revealed the tension between the duty to care for the sick child and the need to care for the other child, creating a conflict between the expectation of balanced motherhood and the reality of an emergency, affecting the bond with children and weakening the family support network due to feelings of neglect and loneliness among family members.

Freire’s approach emphasizes certain fundamental concepts and procedures to strengthen a clear connection between the weaknesses of family relationships and the importance of activities that promote communication and affection. Among the defining concepts are dialogue and loving kindness, which are actions that encourage family members to take responsibility for outcomes, whether positive or negative. By promoting dialogue and loving kindness, families can recognize the factors that cause distancing and isolation in caregivers, reestablishing fragile connections and creating a more welcoming and supportive environment, ensuring support for those involved^(9)^.

In addition to the therapeutic process of a child with cancer influencing social relationships, it also interferes with the family’s livelihood due to the extra expenses arising from the treatment^(32)^. Costs related to treatment, expressed by medications not covered by the Brazilian Health System, such as transportation, food, accommodation for mothers in the city where treatment is being carried out, in the case of those who come from other regions, among other factors, end up overloading the family budget, which can lead to financial toxicity, which is a concept that describes the stress and continuous concern with the expenses related to cancer treatment^(33)^.

Caregiving mothers’ discourses present them as subjects subjected to material (economic) and ideological conditions, with the situation of illness imposing a regime of sacrifice and scarcity, where mothers often abandon material possessions and renounce tasks, positions, and jobs to dedicate themselves fully to their children’s health. This fact symbolizes the transformation of their identity and relationship with daily life, reflecting how much their children’s health has become the center of their concerns and actions^(34)^.

Freire’s method^(9)^ emphasizes the fight against oppression and the pursuit of education that promotes the humanization of individuals. The discourses analyzed reveal a scenario of economic oppression, where families face economic vulnerability and depend on their own survival capabilities. Oppression dehumanizes individuals, forcing them to make decisions that restrict their freedom and potential. However, even in adverse contexts, it is possible to promote transformative actions through organization and collective awareness.

In participants’ discourses, we can see “limit-situations” that symbolize unstable moments in which caregiving women-mothers find themselves confronted with challenging circumstances, which demand reflection as an opportunity to transform reality. These situations should be faced not with the prospect of insurmountable barriers, but rather with the alternative of new actions, paths, and changes, establishing the “unheard-of-viable”^(35)^.

“Limit-acts” are actions taken to break out of “limit-situations”, and, in the case of mothers of children with cancer, these actions can be driven by education and dialogue, allowing them to seek information about cancer treatment and demand better healthcare conditions, both for the child and for the family^(36)^. In this process, building support networks, including family, friends, support groups, and healthcare professionals such as psychologists, is essential. These strategies provide social, psychological, emotional, and practical support, helping to reduce the burden and stressors faced by many mothers^(35)^.

At this core, caring for their health is a key point in the process of their children’s illness and treatment, given that they abandon their own identity for children’s needs, taking on the aspects that permeate cancer therapy. Therefore, promoting healthcare through interdisciplinarity should be seen as a priority for caregivers during their children’s cancer treatment, as caring for their health involves recognizing their own physical, emotional, psychological, and social needs, and is essential for mothers to remain healthy and resilient in the face of adversity^(37)^.

### Study limitations

The limitations of this study are expressed by the limited sample of participants, which may not reflect the diversity of experiences and opinions of the target audience. On the other hand, the specificity of the studied context may restrict the generalization of results to other settings and, thus, limit the applicability of research findings to different environments.

### Contributions to nursing and health

Nursing professionals play a fundamental role in this line of care, as they must have the ability to listen, taking into account the previous experiences of these caregivers and promoting a liberating education, with care and assistance guidance for both patients and caregivers based on dialogue. Hence, the author proposes that nurses adopt intentional communication and sensitive listening to reduce the anguish and stress that treatment generates for the mother-child dyad. Furthermore, a space for dialogue should be offered so that they can express their feelings and prepare for the changes based on the experiences they have experienced during their children’s cancer treatment.

## FINAL CONSIDERATIONS

This study allowed us to understand the experiences of women-mothers caring for children with cancer, highlighting the emotional, psychological, and physical impacts, as well as repercussions on social and family dynamics and potential personal care strategies, based on the applicability of CSM and Paulo Freire’s theoretical framework. Hence, sensitive listening proved essential for them to express their experiences, anxieties, and feelings, often silenced by the demands of continuous child care.

The research highlighted the numerous challenges faced by the participants, who in most cases find themselves overwhelmed and distress, requiring support from their social support network, as well as care practices based on humanization, dialogue, and liberating education. It is also important to note the importance of these women being heard and welcomed comprehensively in their physical, psychological, emotional, and social dimensions.

Thus, it is necessary for healthcare professionals, especially nurses, to advocate for a holistic and comprehensive approach, in which women-mothers who are caregivers are seen in all their dimensions—physical, psychological, emotional, social, and family. This fact is recognized in mothers’ discourse, in which they acknowledge that medical care alone is not sufficient, emphasizing issues involving psychological support to assist with emotional and mental aspects, which is consistent with Freire’s vision of education (or care) that embraces the human being in their entirety.

## Data Availability

The research data are available only upon request.
