# Supplementary material for: Sensitive listening of women-mothers caring for children with cancer about self-care
Source: Rev Bras Enferm. 2026 Jan 9;78(Suppl 3):e20250027. doi: 10.1590/0034-7167-2025-0027 (PMC12798865; doi:10.1590/0034-7167-2025-0027)
Supplement: Translation

# Translation: Escuta sensível de mulheres-mães cuidadoras de criança com câncer acerca do cuidado de si

**DOI:** 10.1590/0034-7167-2025-0027pt

**Authors:** Dociana Erica Cabral Formigosa, George Pinheiro Carvalho, Lucrecia Aline Cabral Formigosa, Hosana de Nazaré Miranda de Carvalho, Joana Dulce Cabral Formigosa, Renato da Costa Teixeira, Marcia Helena Machado Nascimento, Maria Goreth Silva Ferreira

**Affiliations:** I Universidade do Estado do Pará. Belém, Pará, brasil.; II Universidade do Estado do Pará. Santarém, Pará, brasil.

**Keywords:** Maternidades, Criança, Autocuidado, Neoplasias, Cuidados de Enfermagem

## Abstract

**Objetivos:**

compreender as vivências de mulheres-mães cuidadoras de crianças com câncer sobre o cuidado de si.

**Métodos:**

pesquisa descritiva, qualitativa, utilizando o Método Criativo Sensível. A coleta de dados ocorreu no ambulatório de hospital oncológico infantil em Belém, Pará, com 16 participantes, através de dinâmicas de criatividade e sensibilidade em dois encontros, os quais foram gravados, transcritos e analisados conforme a análise de discurso francesa, fundamentando-se na base teórica de Paulo Freire.

**Resultados:**

emergiram três temas principais: 1) “Impactos na saúde da mulher-mãe cuidadora”, com subtemas de impactos emocionais, psicológicos e físicos; 2) “Dedicação ao cuidado da criança e seu impacto na dinâmica familiar”, incluindo o subtema sobre relações familiares; 3) “Apoio institucional e políticas públicas”.

**Considerações Finais:**

as mulheres-mães cuidadoras enfrentam impactos significativos em sua saúde física e emocional, e dinâmicas familiares, sendo necessária a implementação de estratégias de cuidado que considerem suas vivências, melhorando sua qualidade de vida.

## INTRODUÇÃO

O câncer é uma doença tão antiga quanto a humanidade, sendo reconhecido como um dos principais desafios de saúde global, levantando preocupações significativas em relação à prevenção, diagnóstico precoce, tratamento e reabilitação. Dados recentes indicam que, em países com baixo Índice de Desenvolvimento Humano (IDH), projeta-se um aumento de 112% nos casos de câncer, enquanto países com IDH médio podem apresentar crescimento de 86% entre 2012 e 2035^(1)^.

No Brasil, para o triênio 2023-2025, devem ser registrados cerca de 704 mil casos novos da doença, quando se exclui o câncer de pele não melanoma. Entre esses casos, 7.930 ocorrerão em crianças e adolescentes de 0 a 19 anos, com uma distribuição de 4.230 casos no sexo masculino e 3.700 no sexo feminino. Nesse contexto, destacam-se as maiores taxas de incidência de câncer, com 3.310 novos casos na região Sudeste e 2.130 na região Nordeste, enquanto as regiões Sul, Centro-Oeste e Norte terão estimativas significativamente menores, com 1.180, 660 e 650 casos, respectivamente^(2)^.

Na internação de pacientes pediátricos, a permanência do acompanhante é necessária, e os estabelecimentos de saúde devem se adequar à presença dos pais ou responsáveis durante o tempo de hospitalização, garantindo-lhes acesso igualitário^(3)^. Em geral, é a mãe quem mais acompanha o filho no processo de adoecimento e hospitalização, sendo uma experiência emocional e fisicamente desgastante para as mulheres-mães cuidadoras, podendo levar a inúmeros problemas de saúde se não houver suporte adequado^(4)^.

Neste estudo, o termo “mulheres-mães cuidadoras” foi empregado como forma de refletir o complexo papel de uma mulher que, além de cuidar de seus filhos de forma tradicional, assume responsabilidades significativas e frequentemente desafiadoras relacionadas a condições de saúde graves como o câncer. Esse papel multifacetado exige um suporte abrangente e uma abordagem sensível para atender, de maneira eficaz, às necessidades dessas mulheres^(5)^.

A literatura aponta que o impacto do diagnóstico e tratamento do câncer infantil transcende a criança, afetando significativamente as mulheres-mães cuidadoras pela sobrecarga física, psicológica, emocional e social. Sabe-se que a incerteza em relação ao tratamento e prognóstico da criança, associada aos sentimentos de impotência, tristeza profunda e exaustão física e emocional, pode favorecer o desenvolvimento de quadros depressivos, repercutindo diretamente em diversos aspectos de saúde das mulheres-mães cuidadoras, tais como distúrbios do sono, alterações no padrão alimentar, fadiga intensa, comprometimento imunológico, além de gerar impactos negativos em sua vida social, afetiva e relacional^(6)^.

Todavia, persistem lacunas na abordagem integral dessas mães no contexto do tratamento oncológico infantil, o que justifica a necessidade deste estudo, tendo como elemento central compreender as repercussões desse processo na vida dessas mulheres, com ênfase nas formas como percebem e vivenciam o cuidado de si. Nesse sentido, é imprescindível considerar que a saúde das mulheres-mães cuidadoras constitui um elemento central para a qualidade do cuidado prestado à criança em tratamento oncológico. Por isso, é essencial que ela também receba atenção e suporte durante toda a jornada de tratamento de seu filho. Tal compreensão busca subsidiar práticas de cuidado e suporte multiprofissional mais sensíveis, além de contribuir para a formulação de intervenções que considerem as necessidades e especificidades dessas mães, visando à melhoria de sua qualidade de vida e, consequentemente, da criança em tratamento^(7,8)^.

## OBJETIVOS

Compreender as vivências de mulheres-mães cuidadoras de crianças com câncer sobre o cuidado de si.

## MÉTODOS

### Aspectos éticos

A pesquisa respeitou os preceitos éticos da Resolução nº 466/2012 do Conselho Nacional de Saúde, que regulamenta as pesquisas envolvendo seres humanos, sendo aprovado pelo Comitê de Ética em Pesquisa da Escola de Enfermagem “Magalhães Barata” da Universidade do Estado do Pará. O consentimento ético das participantes foi adquirido mediante a assinatura do Termo de Consentimento Livre e Esclarecido. Como forma de manter o anonimato de identidade, estas foram identificadas, no decorrer do texto, por códigos alfanuméricos (M1...M16).

### Referencial teórico

A investigação das experiências das mulheres-mães cuidadoras foi fundamentada no referencial teórico crítico-reflexivo de Paulo Freire, operacionalizada pelo Método Criativo Sensível (MCS), uma vez que este favorece a escuta sensível, dialogicidade e construção coletiva do conhecimento, permitindo compreender profundamente os significados e sentidos atribuídos pelas participantes às suas vivências, considerando os aspectos biopsicossociais^(9-11)^.

Utilizou-se como referencial teórico a abordagem crítica e reflexiva de Paulo Freire, a qual institui a prática educativa como um ato de libertação dos indivíduos perante as amarras opressoras da sociedade, permitindo que eles se tornem agentes ativos na construção do conhecimento e mudança de dada realidade. A singularidade do método Paulo Freire desponta em questões relacionadas à conscientização do sujeito, em que se tem uma nova visão da realidade provocada pela criticidade, além da consciência comunitária para lidar com situações-limite comuns^(9,10)^.

A teoria de Paulo Freire enfatiza que o diálogo tem papel de destaque no processo educativo, o qual, para ser autêntico, deve ser pautado na igualdade e respeito mútuo, com participação ativa e reflexiva do educando e do educador, na busca da ação e emancipação. Assim, a dialogicidade interligada à amorosidade deve estar presente em todos os momentos desse processo, desde a escolha do conteúdo até temas geradores e método, o que torna possível a construção de uma pedagogia ética, social e crítica capaz de trazer a conscientização libertadora da opressão ainda vigente na sociedade^(9)^.

### Tipo de estudo

Trata-se de pesquisa descritiva, com abordagem qualitativa, com base no MCS, o qual está fundamentado na tríade discussão de grupo, observação dos participantes, e dinâmica de criatividade e sensibilidade^(11)^. Neste sentido, o método se adequa ao objeto de estudo por se fundamentar no princípio da dialogicidade, que enfatiza a inseparabilidade do ser humano de sua história e reconhece a linguagem como um instrumento essencial de interação social^(12)^. Ademais, o referencial crítico-reflexivo de Paulo Freire permite que os participantes expressem suas ideias e opiniões livremente, promovendo uma prática desopressora mediada pela liberdade de pensamento, estimulando a criatividade e múltiplas formas de manifestação, especialmente os sentidos do corpo e a sensibilidade, por meio de dinâmicas interativas^(9,13)^.

### Procedimentos metodológicos

#### Cenário de estudo

O estudo foi realizado no ambulatório de hospital oncológico infantil localizado no município de Belém, estado do Pará. A escolha do local se deu por se tratar de uma Unidade de Alta Complexidade em Oncologia e único hospital público do Pará a oferecer tratamento especializado para câncer em crianças e adolescentes de 0 a 19 anos^(14)^.

#### Participantes

Participaram da pesquisa 16 mulheres-mães cuidadoras de crianças diagnosticadas e em tratamento de câncer no ambulatório do hospital. Como critérios de inclusão, consideraram-se as mães de crianças diagnosticadas com câncer na faixa etária de 0 a 11 anos, 11 meses e 29 dias^(15)^, e em regime de tratamento há pelo menos seis meses no hospital de estudo. Foram excluídas aquelas com idade inferior a 18 anos de idade, que não eram alfabetizadas ou que possuíam déficit cognitivo e/ou comorbidades que inviabilizavam a coleta de dados.

#### Produção de dados

A produção de dados ocorreu no período de janeiro a fevereiro de 2024, por meio de dinâmicas de criatividade e sensibilidade, que se apresenta como eixo central do MCS. As participantes foram divididas, de forma aleatória, em dois subgrupos de oito mulheres-mães cuidadoras em cada, sendo elencadas as dinâmicas árvore do conhecimento e corpo-saber para efetivação da coleta de dados. Os encontros foram gravados em formato de vídeo e áudio em aparelhos do tipo *smartphone*, sendo, posteriormente, transcritos.

Para a operacionalização das dinâmicas, foi organizado cinco passos, a saber: 1) organização do ambiente e material a ser utilizado nas produções artísticas; 2) apresentação do objetivo da pesquisa e leitura do Termo de Consentimento Livre Esclarecido e Termo de Uso de Imagem e Som, além do preenchimento da ficha de caracterização sociodemográfica; 3) explanação da dinâmica e seus objetivos, bem como a distribuição dos materiais para construção artística e apresentação da questão geradora do debate; 4) exposição da produção artística individual, seguida da coletivização das experiências individuais; 5) análise coletiva, síntese das experiências e validação dos dados. Vale ressaltar que as atividades foram realizadas por um pesquisador principal, dois pesquisadores assistentes e um profissional psicólogo, objetivando assegurar apoio psicológico àquelas que necessitassem.

A dinâmica árvore do conhecimento é baseada na produção artística coletiva de uma árvore, na qual os participantes estabelecem analogias entre as partes que a compõem e o objeto de pesquisa proposto pelo sujeito-pesquisador, considerando também a questão geradora do debate^(16)^. Configura-se em abordagem que favoreceu a dialogicidade entre os participantes, revelando os significados do processo de cuidado de si, associados às experiências vivenciadas durante o diagnóstico e tratamento do filho. A questão geradora do debate foi formulada da seguinte maneira: assim como a árvore precisa dos elementos da natureza para crescer forte e saudável, produzindo flores e frutos, o que você considera importante para o processo de cuidar de si durante o tratamento de seu filho?

A dinâmica corpo-saber é desenvolvida com a produção artística pelos participantes, com uso determinados recursos, tais como cartazes, lápis de cor e canetas, sendo mediada pelo desenho de um corpo humano para relacionar aos cuidados de saúde, seja consigo ou com os outros^(17)^. O desenvolvimento da atividade ocorreu a partir da seguinte questão geradora de debate: de que maneira o processo de adoecimento e hospitalização da criança reflete no seu corpo e na sua saúde?

#### Análise de dados

A análise do material transcrito foi realizada com base na análise de discurso da linha francesa, utilizando os textos gerados nas dinâmicas de criatividade e sensibilidade. Trata-se de metodologia sustentada pela materialidade linguística, a partir da exploração da produção de sentidos durante a leitura, permitindo captar significados que vão além das palavras, considerando pausas e interrupções que afetam o discurso e se interligam às tensões emocionais e cognitivas presentes nas narrativas^(18)^. Ademais, esta análise encontra-se dividida em três etapas: pré-análise discursiva; análise discursiva; e pós-análise discursiva^(19)^.

Na pré-análise discursiva, ocorreu a definição do *corpus* textual de acordo com os discursos das mulheres-mães cuidadoras. A análise discursiva empregou quatro fases, expressas pela transcrição dos dados com leitura flutuante e identificação dos temas, análise do objeto discursivo, em que há o entendimento dos sentidos originados, análise do processo discursivo, com a atribuição de sentidos a partir da relação das atividades discursivas verbal e não verbal, e elaboração das tipologias discursivas. Por fim, a pós-análise foi fundamentada na contextualização, interpretação e apresentação da análise com base na relação com o referencial teórico de Paulo Freire^(19,20)^.

## RESULTADOS

### Caracterização das participantes

Participaram da pesquisa 16 mulheres-mães cuidadoras, das quais cinco residiam na Região Metropolitana de Belém, e 11 moravam em outros municípios, o que as coloca a uma distância significativa do local onde seus filhos recebem tratamento. A faixa etária variou entre 22 e 47 anos. Em termos de escolaridade, oito mulheres tinham o ensino médio, seguidas por seis com ensino fundamental e dois com ensino superior. O estado civil predominante foi casado, com oito participantes nessa condição, bem como dois se encontravam em união consensual, ao passo que três se designaram como divorciadas e três, solteiras. A maioria das participantes, totalizando 11, não tinha ocupação remunerada, dedicando-se às atividades domésticas, e a renda familiar de dez delas era de apenas um salário mínimo. Quanto aos filhos, a maioria das mulheres tinha dois filhos, com idade média de 7 anos, e o tempo médio desde o diagnóstico foi de três anos.

Os dados são apresentados, inicialmente, por meio das produções artísticas das mulheres-mães cuidadoras, que expressam suas experiências subjetivas no contexto do cuidado oncológico de seus filhos. Em seguida, são discutidos os impactos ocasionados por essa condição em múltiplas esferas da vida dessas mulheres, incluindo os aspectos emocionais, psicológicos, físicos, sociais e familiares, de modo a proporcionar uma compreensão integrada e aprofundada de suas vivências.

### Dinâmica árvore do conhecimento

As participantes desenvolveram em um encontro uma produção artística e verbalizaram em grupo acerca de suas experiências vivenciadas com o diagnóstico e tratamento da criança com câncer. Desse modo, seguindo a questão geradora do debate, elas relataram os elementos que consideravam importantes para o processo de cuidar de si durante o tratamento do filho, conforme a Figura 1.

**Figura 1:** Produção artística realizada pelas mulheres-mães cuidadoras durante a dinâmica árvore do conhecimento, Belém, Pará, Brasil, 2024

### Dinâmica corpo-saber

A partir dessa dinâmica, foi realizada uma produção artística e discutido em grupo sobre os impactos na saúde das mulheres-mães cuidadoras de crianças com câncer e de que forma ocorre o processo de cuidar de si. Com base na questão geradora do debate, as participantes compartilharam suas experiências acerca do processo de saúde-doença no que compete aos sinais de adoecimento e tratamento, como ilustrado na Figura 2.

**Figura 2:** Produção artística realizada pelas mulheres-mães cuidadoras durante a dinâmica corpo-saber, Belém, Pará, Brasil, 2024

### Impactos na saúde das mulheres-mães cuidadoras

As experiências vivenciadas pelas mães no cuidado de seus filhos revelam um cenário complexo, onde as demandas de cuidar de uma criança com câncer impactam profundamente a dinâmica de suas vidas. Além disso, esse impacto é multifacetado, afetando diversas esferas, incluindo a vida pessoal, profissional e emocional das mulheres-mães cuidadoras.

### Impactos emocionais e psicológicos

O processo de cuidar de uma criança com câncer é árduo e intenso, demandando atenção específica, em decorrência das especificidades relativas ao tratamento e suas consequências. As mães lidam constantemente com a sobrecarga de tarefas, especialmente no que diz respeito à assistência integral do filho, o que acaba por refletir no seu processo de cuidado. A análise da fala das participantes revela padrões comuns quanto à omissão do cuidado de si, uma vez que os relatos apontam a prioridade na assistência à criança acima de suas próprias prioridades e saúde, destacando ainda a relação com o cansaço e exaustão.

*Eu, depois que passou por tudo* [doença], *acho que eu passei a ficar mais cansada, mais despreocupada comigo mesmo, sabe?* [...]. (M1)

*Mas minha preocupação maior é com ela, e acabo me deixando mais de lado* [...]. (M2)

[...] *eu acho que, como eu foquei muito tempo em cuidar dela* [filha em tratamento], *a gente acaba se deixando mais de lado* [...]. (M5)

*Nesses quatro anos, o único médico que eu vou é só no ginecologista, isso quando eu sinto algum incômodo* [...]. (M11)

[...] *tipo cuidar, cuidar não. O único cuidado que eu tenho, tipo, é só mesmo pedir pra Deus me proteger* [...]. (M16)

A constante priorização das necessidades da criança reverbera no esgotamento e exaustão não só física, mas também psicológica e emocional das mulheres-mães cuidadoras, resultando no estado de fadiga crônica, que pode levar ao desenvolvimento de distúrbios psiquiátricos. As falas das participantes refletem uma prática discursiva fortemente marcada pelo sofrimento, ansiedade e depressão, que são efeitos da experiência de cuidar de uma criança com câncer. Dessa maneira, as mães constroem suas identidades no discurso como cuidadoras exaustas, sobrecarregadas pela responsabilidade de manter a vida dos filhos.

*Eu também cheguei assim, do meu marido me tirar de uma corda depois do diagnóstico da minha filha* [...]. (M5)

[...] *eu tentei me matar depois do diagnóstico da minha filha*. (M9)

[...] *a gente fica com uma carga de ansiedade muito grande. É o tempo todo você ali como se aquela linha da vida do teu filho fosse arrebentar a qualquer momento. Então, são muitos altos e baixos, e aí acaba realmente descontando na comida. Alguns acabam indo para esse lado; outros ficam mesmo depressivos. Eu fui e voltei da depressão assim rápido, porque ao mesmo tempo eu estava ali no fundo do poço, mas eu sabia que ele precisava de mim. E aí, então, como através da fé, a gente consegue assim* [...]. (M12)

*Eu tenho ansiedade* [...]. (M4)

[...] *eu também tenho isso* [ansiedade]. (M7)

Ademais, a produção gráfica na dinâmica árvore do conhecimento ratificou a importância atribuída à espiritualidade e à fé, destacadas nas falas como recursos essenciais durante o adoecimento do filho. As participantes citaram expressões como “fé em Deus” e “espiritualidade”, revelando que tais elementos atuam como suporte emocional e fortalecem o enfrentamento cotidiano. Por outro lado, as participantes destacaram a influência da rede de apoio, incluindo a “Casa de Apoio”, familiares e equipe de saúde como elementos que contribuem para minimizar os impactos do cuidado intensivo à criança em tratamento oncológico.

### Impactos físicos

O diagnóstico da criança gera um desgaste físico significativo para as mulheres-mães cuidadoras, que assumem a responsabilidade principal pelo cuidado do filho doente. As falas das participantes revelam como o tratamento impacta suas demandas físicas, frequentemente negligenciadas em prol do bem-estar da criança. Essas expressões estão inseridas em um contexto de saúde e doença, refletindo em ideologias relacionadas ao corpo e à feminilidade. Ademais, a subjetividade das mães é moldada pela experiência de adoecimento, revelando um eu fragilizado que enfrenta as mudanças corporais e emocionais provocadas pelo tratamento do filho.

[...] *sinto muita dor de cabeça, enxaqueca*. (M6)

[...] *eu engordei. Quando ela começou o tratamento dela, eu pesava 48* [Kg]*. Hoje eu estou com 60* [Kg]. (M12)

[...] *quando eu fico estressada, ansiosa com alguma coisa, começam a aparecer as manchinhas roxas*. (M15)

[...] *meu cabelo era bem grande, caiu muito, cortei, mas não consegui recuperar, porque parece que quando ela faz quimio principalmente, parece quando chego em casa que eu meto a mão, nossa, sai um monte de cabelo*. (M16)

[...] *infelizmente, tem um monte de coisa pra fazer em casa, e a gente não tem tempo pra nada. Quando acaba de fazer*, já é dez, 11 horas da noite. Tem que dormir, porque principalmente eu venho três, quatro vezes na semana com ela pro hospital, então não tenho muito tempo para mim. (M16)

### Dedicação ao cuidado da criança e seu impacto na dinâmica familiar e social

O debate mediado pela produção artística desenha um cenário de como as mulheres enfrentam desafios significativos em decorrência das demandas de cuidados diários da criança, deparando-se com fatores relacionados à migração, vivências familiares e profissionais, aspectos culturais, linguística, crenças religiosas, e questões financeiras. Cada dimensão dessas é refletida por estruturas e processos sociais que moldam as experiências maternas no processo de tratamento do filho, sendo essencial compreender os impactos dessa realidade e as influências em sua saúde e bem-estar, bem como entender as potenciais estratégias adotadas para lidar com as experiências vivenciadas.

### Relações familiares

Com base nos discursos, constatou-se que os cuidados com as crianças doentes afetam as dinâmicas familiares, incluindo o relacionamento com cônjuge e outros filhos e familiares próximos, além dos impactos financeiros. A prática discursiva das mulheres-mães cuidadoras desvela como o tratamento hospitalar de um filho interfere na sua vida cotidiana e nas relações familiares, tendo uma formação discursiva atravessada por formações ideológicas da maternidade, do sacrifício e culpa, assim como a sobrecarga de atividades exercidas por elas, reforçando a perspectiva social que valoriza abnegação das mães.

[...] *meu outro filho* [irmão da criança em tratamento] *reclama muito disso. Ele fala: “Mãe, a senhora não dá atenção pra mim”*. (M16)

*Meu outro filho também reclama muito. Ele fala: “Mãe, a senhora dá muito mais atenção para ela* [criança em tratamento]. *Só é ela, hospital e ela, é para casa e ela”. Ele só vai para a escola, tipo, ele não sai porque eu não tenho muito tempo com ele*. (M2)

Além de influenciar as relações sociais, o processo terapêutico da criança com câncer também impacta o sustento da família devido às despesas extras decorrentes do tratamento^(32)^. Os custos relativos ao tratamento, expressos por medicamentos não cobertos pelo Sistema Único de Saúde, como transporte, alimentação, estadia das mães na cidade de realização do tratamento, no caso daquelas que são oriundas de outras regiões, entre outros fatores, acabam por sobrecarregar o orçamento familiar, podendo acarretar a toxicidade financeira, a qual figura como um conceito que descreve o estresse e a inquietação contínua com as despesas relativas ao tratamento do câncer^(33)^.

[...] *mas eu literalmente abandonei tudo e vendemos tudo o que tinha. Vendemos casa, vendemos carro, tudo que era possível para esse período eu não ter que voltar a trabalhar para conseguir dar continuidade no tratamento, que apesar de ser pelo SUS, mas os nossos gastos são muitos* [...]. (M5)

[...] *e teve aperto, por questões financeiras. A gente, na verdade, ficava com o que sobrava, e era tudo pra ele* [...]. (M9)

### Apoio institucional e políticas públicas

Os discursos das mães refletem seu sofrimento e vulnerabilidade, frequentemente expressando um pedido de apoio que pode ser interpretado como uma reivindicação por políticas institucionais e públicas de saúde que reconheçam o impacto emocional da doença dos filhos na saúde das mães, sendo fundamental para promover o bem-estar delas, o que, por sua vez, melhora a qualidade de vida durante o tratamento dos filhos. Ressalta-se que a saúde mental das mães não afeta apenas suas próprias condições de vida, mas também a qualidade do cuidado que oferecem às crianças.

[...] *às vezes, até aqui no hospital a gente é taxada de estressada, mas não é. A gente já passa por tantas situações. Parece que as pessoas assim não têm empatia, não se colocam no lugar*. (M3)

[...] *eu acho que se deveria sim ter um acompanhamento psicológico com as mãezinhas, entendeu? Eu acho que ajudaria muito*. (M1)

## DISCUSSÃO

A polifonia do discurso das mulheres-mães cuidadoras revela que, no adoecimento do filho, elas se dedicam integralmente ao cuidado da criança, colocando suas próprias necessidades em segundo plano. Nessa jornada de dedicação extrema com seus filhos, elas negligenciam a própria saúde, refletindo um processo de autoesquecimento, em que o bem-estar da mãe fica subjugado às demandas do filho. Desse modo, ao ocupar o lugar de mãe cuidadora, essas mulheres aguentam a dor e dedicam-se constantemente à criança, o que as impede de reconhecer que também precisam de cuidados^(21)^.

A autonegligência, presente nos discursos, indica mudanças na identidade e percepção de si durante o processo de cuidar do outro, impactando a autoimagem e autoestima da mulher, bem como o seu estilo de vida e vulnerabilidade a agravos de saúde^(22)^. A imersão na dinâmica de cuidado do filho hospitalizado é marcada por responsabilidades e incertezas que geram sensação de risco constante, alinhando-se ao pensamento de Freire, pois as mães não encontram espaço para se afastar da situação em que estão inseridas e não conseguem se distanciar para refletir criticamente sobre sua condição, não ocorrendo, consequentemente, a alteração de paradigma^(23)^.

O cotidiano dessas mães é marcado por estresse contínuo, preocupação constante e falta de descanso, o que impacta negativamente suas condições físicas e qualidade de vida^(24)^. As participantes relatam sintomas como dor de cabeça, enxaqueca e hematomas, ilustrando a associação entre o estresse e as reações do organismo, refletindo a sobrecarga mental e emocional que enfrentam^(25)^. Além disso, as mães mencionam mudanças físicas negativas, como ganho de peso e queda de cabelo relacionada aos medicamentos quimioterápicos, que afetam sua autoestima e autoimagem, influenciadas por padrões sociais de beleza e feminilidade^(26)^.

As formações discursivas denotam tentativas de suicídio resultantes do sofrimento extremo e exaustão, refletindo o grau crítico de dor e desespero vivenciado diante do diagnóstico da doença de seus filhos. A linguagem empregada pelas mulheres reflete a ruptura em sua subjetividade, pois o câncer carrega o estigma de ser uma sentença de morte, significando para elas um desfecho trágico para a criança, levando ao sofrimento e graves crises psicológicas e emocionais^(27)^.

Os sintomas de distúrbios psiquiátricos refletem o impacto significativo na saúde física e mental, qualidade de vida e sistema imunológico do cuidador, exacerbando a possibilidade do desenvolvimento de comorbidades, sejam elas agudas ou crônicas. Além do mais, o adoecimento do cuidador implica o déficit da qualidade do cuidado prestado, bem como a continuidade das atividades desenvolvidas nos âmbitos pessoal, familiar e/ou profissional^(28)^.

Os achados indicam que, em determinados momentos, as participantes usam a religiosidade e espiritualidade como estratégia de amenizar o sofrimento, controlar as emoções e encontrar forças durante o tratamento de um filho com câncer. A religiosidade auxilia no alívio do estresse e equilíbrio psicológico, funcionando como um mecanismo de enfrentamento e um alicerce emocional. Nesse sentido, é fundamental a padronização nas orientações e práticas do cuidado, além da implementação de estratégias de enfrentamento aos distúrbios psiquiátricos e psicossomáticos, objetivando tornar os cuidadores mais capacitados e menos sobrecarregados, acarretando na diminuição do impacto negativo em sua saúde e bem-estar^(29)^.

A partir dos discursos das participantes, identifica-se o diálogo como forma de fomento à prática de cuidado, sendo uma ferramenta de conscientização e transformação social que propõe a educação como uma prática reflexiva e libertadora, fundamentada na empatia e no reconhecimento mútuo de vivências, denominada de educação libertadora. Essa abordagem incentiva a busca por apoio, organização comunitária e fortalecimento de laços para combater a opressão emocional e social. Outrossim, o diálogo com as mães permitiu a expressão de suas emoções, medos e inseguranças relacionadas ao tratamento da criança, promovendo o reconhecimento de suas próprias necessidades e a busca da melhor maneira para cuidar de si mesmas enquanto cuidam dos filhos^(9)^.

Nesse contexto, o enfermeiro detém papel primordial no processo de superação da opressão, promovendo estratégias de cuidado que favoreçam a humanização do cuidado e construção de vínculos terapêuticos de confiança com base nos preceitos de Freire que levem as mulheres-mães cuidadoras a reconhecerem sua própria condição e que possam agir para transformá-la. Em outras palavras, a escuta sensível e o diálogo surgem como estratégias de sensibilização que podem levá-las a repensar o equilíbrio entre cuidar do outro e cuidar de si, reconhecendo a importância do próprio bem-estar e permitindo que elas sejam valorizadas integralmente^(30)^.

A dinâmica familiar é completamente afetada em decorrência do tratamento da criança com câncer, uma vez que a mãe se dedica às atividades interligadas ao filho doente, tais como acompanhamento no hospital e internação, consultas médicas, administração de medicamentos, entre outros, que podem levar ao afastamento das outras demandas familiares, sociais e conjugais^(31)^. Os discursos mostraram a tensão entre o dever de cuidar do filho doente e a necessidade de atender ao outro filho, criando um conflito entre a expectativa de uma maternidade equilibrada e a realidade de uma emergência, afetando o vínculo com os filhos e fragilizando a rede de apoio familiar por conta dos sentimentos de negligência e solidão entre os membros da família.

A abordagem de Freire estima determinados conceitos e procedimentos fundamentais para fortalecer uma conexão clara entre as fragilidades das relações familiares e a importância de atividades que promovam comunicação e afeto. Entre os conceitos determinantes, há diálogo e amorosidade, os quais se configuram como ações que instigam os indivíduos da família a assumirem responsabilidades pelos resultados, sejam eles positivos ou negativos. Ao promover o diálogo e a amorosidade, as famílias podem reconhecer os fatores que causam distanciamento e isolamento das mulheres-mães cuidadoras, restabelecendo as conexões fragilizadas e criando um ambiente mais acolhedor e solidário, garantindo suporte aos envolvidos^(9)^.

Além do processo terapêutico da criança com câncer influenciar as relações sociais, este interfere no sustento da família por conta das despesas extras decorrentes do tratamento^(32)^. Os custos relativos ao tratamento, expressos por medicamentos não cobertos pelo Sistema Único de Saúde, como transporte, alimentação, estadia das mães na cidade de realização do tratamento, no caso daquelas que são oriundas de outras regiões, entre outros fatores, acabam por sobrecarregar o orçamento familiar, podendo acarretar a toxicidade financeira, a qual figura como um conceito que descreve o estresse e a inquietação contínua com as despesas relativas ao tratamento do câncer^(33)^.

Os discursos das mulheres mães e cuidadoras as apresentam como sujeitos submetidos a condições materiais (econômicas) e ideológicas, com a situação de doença impondo um regime de sacrifício e escassez, onde as mães frequentemente abandonam bens materiais e renunciam a tarefas, cargos e empregos para se dedicar integralmente à saúde da criança. Tal fato simboliza a transformação da identidade e relação com a vida cotidiana, refletindo o quanto a saúde do filho tomou o centro de suas preocupações e ações^(34)^.

O método Paulo Freire^(9)^ destaca a luta contra a opressão e a busca pela educação que promova a humanização dos indivíduos. Os discursos analisados revelam um cenário de opressão econômica, onde as famílias enfrentam vulnerabilidades econômica e dependem de suas próprias capacidades de sobrevivência. A opressão desumaniza os indivíduos, forçando-os a tomar decisões que restringem sua liberdade e potencial; no entanto, mesmo em contextos adversos, é possível promover ações transformadoras através da organização e conscientização coletiva.

Nos discursos das participantes, percebem-se as “situações-limite” que simbolizam momentos instáveis em que as mulheres-mães cuidadoras se veem confrontadas por circunstâncias desafiadoras, as quais demandam de reflexão como oportunidade de transformar a realidade. Essas situações devem ser enfrentadas não com a perspectiva de barreiras insuperáveis, mas sim com a alternativa de novas ações, caminhos e mudanças, instaurando o “inédito-viável”^(35)^.

Os “atos-limite” são ações tomadas para romper “situações-limite”, e, no caso das mães de crianças com câncer, esses atos podem ser impulsionados pela educação e pelo diálogo, permitindo-lhes buscar informações sobre o tratamento oncológico e reivindicar melhores condições de assistência à saúde, tanto para a criança quanto para a família^(36)^. Nesse processo, a construção de redes de apoio, incluindo familiares, amigos, grupos de apoio e profissionais de saúde como psicólogos, é essencial. Essas estratégias proporcionam suporte social, psicológico, emocional e prático, ajudando a reduzir a sobrecarga e os estressores enfrentados por muitas mães^(35)^.

Nesse âmago, o cuidado de sua saúde é um ponto-chave no processo de adoecimento e tratamento do filho, tendo em vista que elas abandonam a própria identidade em razão das necessidades da criança, tomando para si os aspectos que permeiam a terapêutica oncológica. Portanto, a promoção do cuidado de sua saúde por meio da interdisciplinaridade deve ser vista como prioridade diante do cuidador durante o tratamento oncológico da criança, pois o cuidado de sua saúde envolve o reconhecimento das suas próprias demandas físicas, emocionais, psicológicas e sociais, sendo fundamental para que as mães possam se manter saudáveis e resilientes diante das adversidades^(37)^.

### Limitações do estudo

As limitações deste estudo são expressas pela amostra restrita de participantes, que pode não refletir a diversidade de experiências e opiniões do público-alvo. Por outro lado, a especificidade do contexto estudado pode restringir a generalização dos resultados para outros cenários e, assim, implicar a limitação de aplicabilidade dos resultados obtidos na pesquisa para ambientes distintos.

### Contribuições para as áreas da enfermagem e saúde

O profissional de enfermagem desempenha um papel fundamental nessa linha de assistência, uma vez que deve ter a capacidade de escuta, levando em consideração as vivências prévias dessas mulheres-mães cuidadoras e promovendo uma educação libertadora, com orientações de cuidados e assistência ao paciente e ao cuidador pautados na dialogicidade. Neste sentido, a autora propõe que o enfermeiro adote a comunicação intencional e escuta sensível para diminuir as angústias e estresses que o tratamento gera ao binômio mãe-criança. Além do mais, propõe que seja oferecido um espaço de diálogo para que possam expressar seus sentimentos e se preparem para as mudanças a partir das experiências vivenciadas durante o tratamento do câncer do filho.

## CONSIDERAÇÕES FINAIS

A realização deste estudo permitiu compreender as experiências das mulheres-mães cuidadoras de crianças com câncer, destacando os impactos emocionais, psicológicos e físicos, além de repercussões nas dinâmicas sociais, familiares e possíveis estratégias de cuidado pessoal, com base na aplicabilidade do MCS e no referencial teórico de Paulo Freire. Nesse sentido, a escuta sensível se mostrou essencial para que elas expressassem suas vivências, angústias e sentimentos, muitas vezes silenciados pelas exigências do cuidado contínuo à criança.

A pesquisa evidenciou os inúmeros desafios enfrentados pelas participantes que, na maioria dos casos, encontram-se sobrecarregadas e em situação de sofrimento, necessitando de suporte por parte da sua rede de apoio social, bem como de prática assistencial pautada na humanização, diálogo e educação libertadora. Nota-se, ainda, a importância dessas mulheres serem ouvidas e acolhidas de forma integral em suas dimensões física, psicológica, emocional e social.

Nesse sentido, faz-se necessário que os profissionais de saúde, especialmente o enfermeiro, defendam a abordagem holística e integral, na qual as mulheres-mães cuidadoras sejam vistas em todas as suas dimensões, sejam física, psicológica, emocional, social e familiar. Tal fato é reconhecido no discurso das mães, em que elas reconhecem que o acompanhamento médico, por si só, não é suficiente, enfatizando questões envolvendo o suporte psicológico para auxiliar nos aspectos emocionais e mentais, o que é coerente com a visão de Freire de uma educação (ou cuidado) que envolve o ser humano em sua totalidade.

## Data Availability

Os dados de pesquisa só estão disponíveis mediante solicitação.
